# The Long-Term Clinical and Radiographic Evolution of Temporomandibular Joint Involvement in Juvenile Idiopathic Arthritis: A Narrative Review and 15-Year Follow-Up Case Report

**DOI:** 10.3390/children13091211

**Published:** 2026-09-08

**Authors:** Evgenia Sp. Gogou, Vasileios Chr. Psarras, Dionysios Andrinopoulos, Michail Ger. Tzakis

**Affiliations:** Clinic of Orofacial Pain Management, School of Dentistry, National and Kapodistrian University of Athens, 2 Thivon Street, 11528 Athens, Greecemtzakis@dent.uoa.gr (M.G.T.)

**Keywords:** juvenile idiopathic arthritis, temporomandibular joint arthritis, temporomandibular joint involvement, orofacial pain, magnetic resonance imaging, occlusal splint, physiotherapy, long-term follow-up

## Abstract

**Highlights:**

**What are the main findings?**

- TMJ involvement progressed structurally despite systemic antirheumatic treatment, while multidisciplinary management achieved sustained clinical and functional improvement.
- Long-term follow-up demonstrated persistent structural TMJ changes, underscoring the potential long-term orofacial impact of JIA.

**What are the implications of the main findings?**

- Clinical remission does not necessarily reflect structural stability of the TMJ in JIA.
- Longitudinal multidisciplinary surveillance of the TMJ and orofacial complex, including imaging, is warranted to detect clinically silent disease progression.

**Abstract:**

**Background/Objectives**: Juvenile idiopathic arthritis (JIA) is the most common chronic rheumatic disease in childhood and may involve the temporomandibular joint (TMJ), sometimes with limited clinical manifestations. This report combines a narrative review of TMJ involvement in JIA with a 15-year follow-up of a patient with JIA-associated TMJ disease. The objectives were to summarize current evidence regarding the clinical assessment and management of TMJ involvement in JIA and to describe the relationship between long-term clinical and structural findings in an individual patient. **Case Report**: A 12-year-old girl with newly diagnosed oligoarticular JIA was referred by the pediatric rheumatologist to the Clinic of Orofacial Pain Management, National and Kapodistrian University of Athens. At referral, TMJ involvement was identified despite systemic antirheumatic treatment. The patient presented with orofacial pain, restricted mouth opening, mandibular deviation, multiple painful orofacial sites, maximum interincisal opening of 26 mm, reduced mandibular excursions, marked masticatory muscle tenderness, and an anterior open bite. Contrast-enhanced magnetic resonance imaging (MRI) demonstrated bilateral active TMJ synovitis, early condylar erosions, and anterior disc displacement, with reduction on the right and without reduction on the left. Management comprised systemic antirheumatic therapy together with a structured physiotherapy program and a maxillary stabilization splint. **Results**: At one-year follow-up, maximum mouth opening had increased to 50 mm, with substantial improvement in mandibular function and symptoms. Fifteen years after the initial TMJ assessment, the patient remained pain-free with satisfactory mandibular function and a maximum mouth opening of 46 mm. Panoramic radiography at long-term follow-up demonstrated bilateral condylar abnormalities, including flattening, osteophyte formation, and intraosseous cystic changes. Because the initial and long-term assessments were performed using different imaging modalities, these findings cannot be directly compared to establish longitudinal structural progression. **Conclusions**: This case illustrates dissociation between favorable long-term clinical and functional status and substantial structural abnormalities identified at follow-up in a patient with JIA-associated TMJ involvement. It also highlights the limitations of relying on clinical symptoms and function alone to characterize TMJ structural status. The literature supports multidisciplinary management and individualized clinical and imaging assessment of TMJ involvement in JIA; however, prospective studies with standardized serial imaging are needed to better define the relationship between clinical outcomes and structural changes over time.

## 1. Introduction

Juvenile idiopathic arthritis (JIA) is a heterogeneous group of chronic inflammatory arthritides with onset before 16 years of age and persistent arthritis for at least six weeks, after exclusion of other causes [1]. Despite advances in diagnosis and treatment, persistent inflammatory activity may result in structural joint damage and functional impairment. Among the joints that may be affected, the temporomandibular joint (TMJ) is of particular concern because inflammation may interfere with mandibular growth and function during a critical period of craniofacial development [2].

TMJ involvement is common in children with JIA and may occur at any stage of the disease. Importantly, TMJ arthritis may remain clinically silent, and the severity of clinical symptoms does not necessarily reflect the extent of underlying joint pathology. Structural changes, including condylar abnormalities and alterations in mandibular growth, may therefore develop in the absence of prominent pain or functional impairment [3]. Magnetic resonance imaging (MRI) is particularly valuable for detecting active inflammatory changes and structural abnormalities that may not be apparent clinically. Accordingly, assessment of TMJ involvement cannot be based exclusively on symptoms or functional findings.

Although the clinical-radiographic dissociation of TMJ involvement in JIA has been previously recognized, long-term longitudinal observations documenting the relationship between sustained clinical and functional outcomes and structural TMJ status remain limited. In particular, there is limited documentation of patients who achieve substantial functional recovery following active TMJ involvement but subsequently demonstrate persistent or progressive structural abnormalities and changes consistent with chronic TMJ involvement.

The aim of this report was to describe the 15-year clinical and radiographic course of TMJ involvement in a patient with JIA, with particular emphasis on the relationship between long-term pain and mandibular function and the structural status of the TMJs. A focused review of the relevant literature was undertaken to contextualize the clinical and imaging findings observed in this case. Written informed consent for treatment and publication of this case report was obtained from the patient’s parents.

## 2. Materials and Methods

### Literature Search

A narrative literature review was conducted using the PubMed/MEDLINE database. The search strategy included combinations of the terms “juvenile idiopathic arthritis” AND “temporomandibular joint”, supplemented by terms related to “magnetic resonance imaging,” “occlusal splint,” “stabilization splint,” “pharmacological treatment,” “orthodontic treatment,” “physiotherapy,” and “long-term outcomes.” Relevant publications addressing the clinical manifestations, diagnosis, imaging, management, and long-term outcomes of TMJ involvement in JIA were reviewed.

JIA is the most common inflammatory rheumatic disease in childhood, with an annual incidence of 8–23 per 100,000 children and a prevalence of approximately 1 in 1000 worldwide [4]. Its development involves complex interactions between genetic susceptibility and environmental factors. According to the International League of Associations for Rheumatology (ILAR) classification, JIA comprises seven subtypes defined by clinical and laboratory features, including the number and pattern of joints involved during the first six months [5]. Immune dysregulation, characterized by increased production of pro-inflammatory cytokines, contributes to persistent synovial inflammation and subsequent joint abnormalities [6].

Clinically, JIA is heterogeneous and may follow a relapsing–remitting course, characterized by joint pain, swelling, morning stiffness, and restricted range of motion. Extra-articular manifestations, particularly chronic anterior uveitis, may be asymptomatic, underscoring the importance of regular screening. Early detection of clinically silent involvement, particularly of the eyes and TMJs, is essential to minimize long-term complications [7].

JIA frequently involves the TMJs, although reported prevalence varies substantially across studies, ranging from 17% to 87%, depending on the patient population, disease duration, JIA subtype, diagnostic criteria, and imaging methodology [8]. Bilateral involvement is common, and structural changes may progress over time, potentially affecting mandibular growth and craniofacial development. TMJ arthritis may also be clinically silent; in one prospective cohort, 71% of children with MRI-detected acute TMJ arthritis were asymptomatic [9]. Therefore, the absence of clinical symptoms does not reliably exclude TMJ involvement, and contrast-enhanced MRI is important for detecting active inflammation and structural changes.

Contrast-enhanced MRI is particularly useful for identifying active TMJ inflammation, including synovial enhancement, joint effusion, and bone marrow edema, whereas chronic structural changes are reflected by condylar deformity, erosion, flattening, osteophyte formation, and growth disturbances [10]. CBCT/CT provides superior assessment of osseous abnormalities and may therefore complement MRI when evaluating chronic structural changes [11]. Conventional radiography, including panoramic imaging, has a more limited role and primarily demonstrates established osseous and growth-related changes rather than active inflammation. Thus, these modalities provide complementary rather than interchangeable information during long-term follow-up. When different imaging techniques are used at different time points, findings should therefore be interpreted cautiously, as apparent changes may reflect differences in modality sensitivity rather than true progression or resolution of disease [12].

The long-term course of TMJ involvement in JIA is characterized by a complex relationship between clinical symptoms, functional findings, and structural changes detected by imaging. Importantly, clinical findings do not always parallel the extent of TMJ abnormalities detected by MRI. In a follow-up study of children with JIA-associated TMJ involvement, clinical symptoms improved in most patients, whereas MRI findings demonstrated persistent or progressive changes, highlighting a discrepancy between clinical improvement and imaging findings [13]. TMJ arthritis may also remain clinically silent, with MRI being more sensitive for detecting active inflammation and structural changes that may not be apparent on physical examination. Therefore, longitudinal imaging, particularly MRI, provides complementary information to clinical assessment and is important for monitoring both inflammatory activity and structural joint changes over time. Even when mandibular function and clinical symptoms improve over time, structural abnormalities of the TMJ may persist or evolve, highlighting a potential discordance between clinical status and imaging findings.

An international multidisciplinary Delphi consensus established standardized terminology distinguishing active TMJ arthritis from TMJ involvement, which encompasses structural and functional consequences, and TMJ symptoms from clinician-observed TMJ dysfunction. Based on this framework, a six-item, consensus-based clinical examination protocol was developed for standardized assessment and longitudinal monitoring of TMJ involvement in children with JIA [14].

Although conventional radiographs may not detect early inflammatory changes, they remain useful for monitoring disease changes and structural sequelae. MRI provides superior visualization of synovial inflammation, cartilage abnormalities, bone marrow edema, and early structural changes, whereas advanced disease may result in joint-space narrowing, erosions, subluxation, or ankylosis [15].

Beyond orofacial manifestations, JIA substantially affects health-related quality of life. Sleep disturbances may further contribute to pain, fatigue, mood changes, reduced physical activity, and poorer quality of life. Consequently, therapeutic intervention aims for the rapid induction and maintenance of clinical remission or minimal disease activity. The primary goal of JIA treatment is sustained remission, characterized by the resolution of clinical symptoms and normalization of disease-related findings. Maintaining remission helps prevent progressive joint destruction, preserve daily functioning, enhance quality of life, and improve long-term outcomes [16]. The 2023 consensus-based recommendations specifically emphasize three major therapeutic objectives in children with JIA and TMJ involvement: (a) control of inflammation, (b) restoration of TMJ function, and (c) normalization of craniofacial growth and prevention of dentofacial changes [17].

Management of TMJ arthritis requires a multidisciplinary approach involving pediatric rheumatologists, dentists, orthodontists, oral and maxillofacial surgeons, radiologists, and orofacial pain specialists. Treatment should be individualized according to disease activity, symptoms, functional impairment, imaging findings, and skeletal maturity. Systemic antirheumatic therapy, including conventional disease-modifying antirheumatic drugs (DMARDs) such as methotrexate and biologic agents when indicated, remains central to controlling JIA-related inflammation [18]. Conservative interventions, including occlusal splints, physiotherapy, and jaw exercises, may contribute to improvements in pain and mandibular function; however, evidence specific to TMJ arthritis in children remains limited and heterogeneous [19]. Orthodontic management in children with JIA and TMJ involvement should be individualized according to disease activity, skeletal maturity, and dentofacial deformity. Functional orthopedic and fixed orthodontic appliances may support mandibular growth and occlusal development, although evidence remains limited [20].

Intra-articular glucocorticoid injections may provide short-term improvement in pain and mandibular opening in selected patients with persistent active TMJ arthritis, although substantial heterogeneity among studies and limited evidence regarding long-term structural and growth outcomes warrant cautious interpretation [21]. Arthrocentesis, with or without corticosteroid injection, may provide symptomatic and functional improvement, but evidence for sustained long-term benefit remains limited. Importantly, clinical improvement, control of active inflammation, and improvement in mandibular function do not necessarily indicate complete resolution of established structural changes. Therefore, long-term clinical and imaging follow-up is essential to distinguish control of inflammatory activity from persistent structural changes and to monitor mandibular growth and function over time [22].

According to the literature reviewed, the present case illustrates a potential dissociation between sustained clinical and functional improvement and long-term structural TMJ changes in JIA. Despite satisfactory pain control and preserved mandibular function, structural abnormalities were identified at long-term follow-up. These findings suggest that clinical improvement alone may not fully reflect the underlying structural status of the TMJ and underscore the importance of continued clinical monitoring and appropriate imaging assessment during long-term management. The clinical course and long-term findings of the present case are described below.

## 3. Case Report

**Figure 1:**
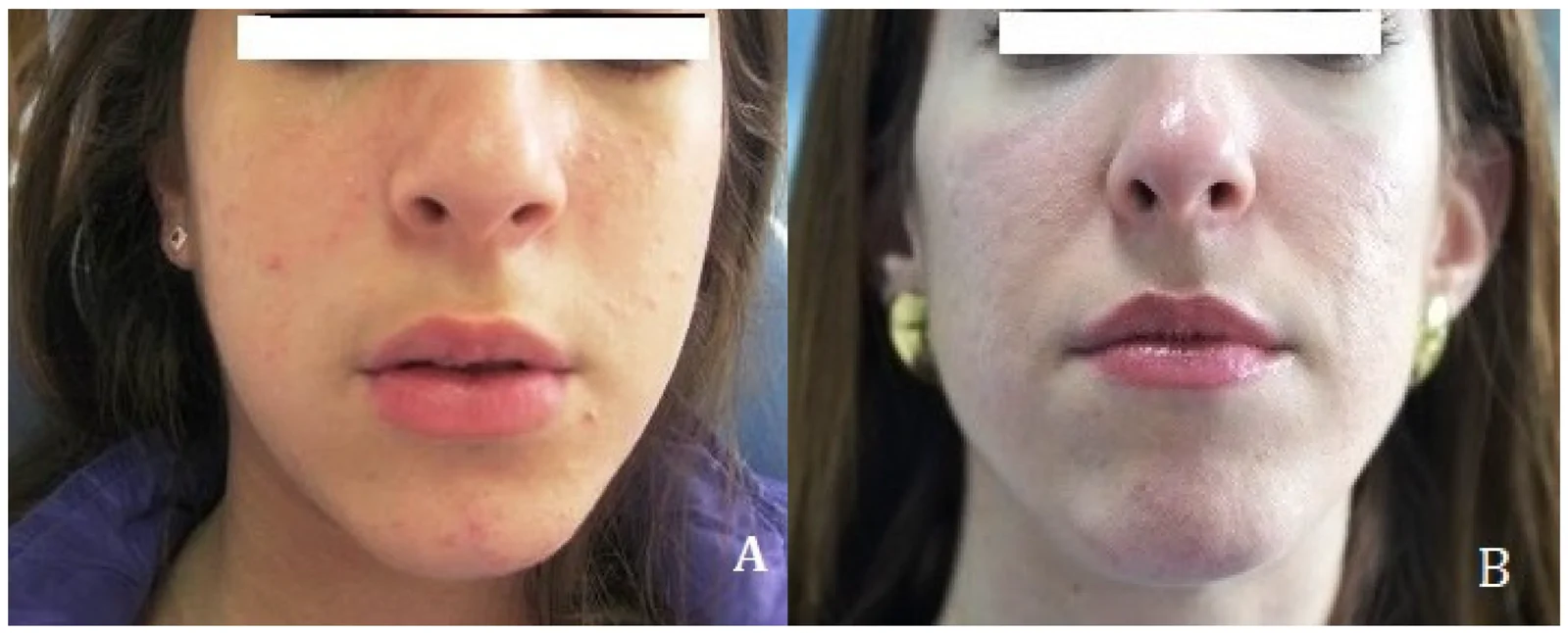
Facial appearance of a female patient with Juvenile Idiopathic Arthritis at two time points. (**A**) At 12 years of age, mild lower facial asymmetry was also apparent, with a slight deviation of the mandibular contour, although the photograph was not acquired under standardized conditions for formal assessment of facial asymmetry. The asymmetry was considered potentially related to asymmetric mandibular growth associated with TMJ involvement. Additionally, mild hypoplasia of the left masseter muscle is evident, possibly related to asymmetric masticatory muscle function. (**B**) At 27 years of age, elongation of the lower facial third persists, and the mild hypoplasia of the left masseter muscle remains evident.

A 12-year-old girl was referred from the Pediatric Rheumatology Outpatient Clinic of the Second Department of Pediatrics, National and Kapodistrian University of Athens, to the Clinic of Orofacial Pain Management, NKUA. The patient was evaluated by the pediatric rheumatologist, and a diagnosis of JIA was established. The referral to the COPM was made for evaluation and management of orofacial pain, restricted mouth opening and mandibular deviation during mouth opening (Figure 1). The patient was diagnosed with oligoarticular JIA at the age of 12 years according to the ILAR classification criteria. TMJ involvement was identified at the same age during the initial orofacial assessment and confirmed by MRI.

MRI of the upper and lower extremities revealed swelling of the left knee with a small amount of joint effusion. In the right hand, a small amount of fluid was observed around the finger tendons at the level of the wrist joint, without evidence of hyperemia in the surrounding tissues. In the left hand, tendon thickening and a small amount of fluid within the wrist joint and along its dorsal aspect were observed, accompanied by marked hyperemia of the surrounding tissues. Furthermore, diffuse thickening and hyperemia of the intra-articular tissues throughout the left wrist joint, extending from the radial to the ulnar aspect, were identified, consistent with an active inflammatory process.

Laboratory evaluation demonstrated a moderately elevated erythrocyte sedimentation rate (ESR) of 18 mm/h (reference range for children: 0–10 mm/h), whereas C-reactive protein (CRP) was 0.6 mg/L. Antinuclear antibodies (ANA) and human leukocyte antigen B27 (HLA-B27) testing were negative. Overall, these findings were consistent with mild systemic inflammatory activity, without serological evidence suggestive of ANA-associated autoimmune disease or HLA-B27-associated arthritis.

Initial pharmacological management included intra-articular corticosteroid injections into the wrist and knee joints, together with systemic oral glucocorticoid therapy. At treatment initiation, prednisolone was administered at 20 mg/day (5 mg, four tablets daily) and was subsequently reduced to 5 mg/day. Methotrexate 2.5 mg tablets (Pfizer, Athens, Greece) was initially administered orally at 8 tablets per week, corresponding to 20 mg/week, assuming a tablet strength of 2.5 mg. The MTX dose was subsequently adjusted to 12.5 mg/week. Adalimumab (Humira^®^, AbbVie, Iraklio, Greece) was also administered during 2011; however, its exact dose and treatment duration could not be reliably established from the available medical records. From 2012, treatment included subcutaneous Methotrexate (Metoject^®^, medac GmbH, Wedel, Germany) at 15 mg/week, together with folinic acid and alfacalcidol. This regimen was continued during the subsequent follow-up, with treatment modifications documented according to the patient’s clinical course.

Two weeks after the diagnosis of JIA, the patient presented to the COPM for the management of restricted mouth opening and pain localized to the left TMJ. The patient reported difficulty opening the mouth, pain during mastication, and deep, pressure-like pain in the left TMJ during functional mandibular movements. Additionally, daily frontal headaches and joint sounds from the left TMJ during jaw movement, also reported.

**Figure 2:**
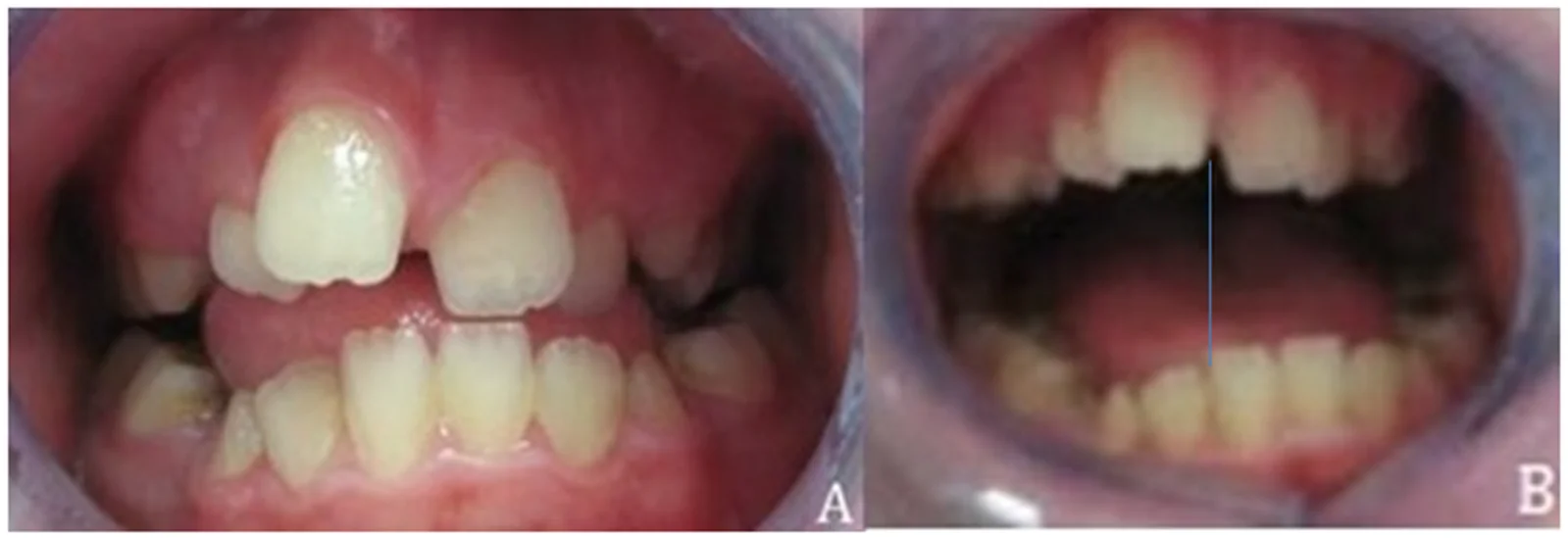
Clinical evaluation of mandibular position in a female patient diagnosed with Juvenile Idiopathic Arthritis at the age of 12 years, shown in closed and open mouth positions. (**A**) In maximum intercuspation, the mandible exhibits occlusal disharmony characterized by an anterior open bite. (**B**) During maximal mandibular opening, a restricted mouth opening of 26 mm is recorded, accompanied by deviation of the mandible toward the affected left side (blue line). These findings indicate possible internal temporomandibular joint derangement, likely associated with anterior disc displacement without reduction.

Clinical examination of the stomatognathic system revealed marked tenderness on bilateral TMJ palpation, with more pronounced pain on lateral palpation and within the external auditory canal on the left side compared with the right. Maximum unassisted interincisal opening was limited to 26 mm (normal ≥ 40 mm). The clinical assessment was performed using a consensus-based short clinical examination protocol, designed for JIA-related orofacial manifestations. The protocol includes 6 items: (1) clinician-assessed pain location; (2) TMJ pain on palpation with the mouth open and closed (unilateral or bilateral); (3) mandibular deviation at maximal mouth opening (>3 mm to the right or left side); (4) maximal unassisted mouth opening in millimeters, accounting for vertical incisal overlap; (5) frontal facial symmetry (presence or absence of asymmetry); and (6) facial profile classification as straight, mildly convex, moderately convex, or micrognathic. Mandibular range of motion during protrusive and lateral excursions was also reduced relative to normal values, indicating impaired TMJ function. Mandibular deviation toward the affected left side was observed during opening (Figure 2). Maximum interincisal opening was assessed by a single examiner, with a total of eight measurements recorded during the period from initiation of treatment in 2011 through 2012. The clinical parameters were recorded during routine clinical assessments at the Clinic of Orofacial Pain Management, with the patient seated and at rest. Maximum interincisal opening was assessed during maximum unassisted mouth opening, while muscle tenderness was evaluated by manual palpation during the clinical examination. Each measurement was obtained using a ruler as the linear distance between the incisal edges of the maxillary and corresponding mandibular central incisors at maximum unassisted mouth opening. The measurements were performed by the same examiner; however, no formal intra-examiner calibration was performed.

Muscle palpation revealed marked tenderness involving masticatory and cervical muscle groups, including the masseter, temporalis, medial and lateral pterygoids, digastric, and sternocleidomastoid muscles. Muscle tenderness was assessed bilaterally by manual palpation and clinically graded as mild, moderate, or severe according to the patient’s response to palpation. Mild tenderness was characterized by minimal or no overt reaction, moderate tenderness by a noticeable but limited pain response, and severe tenderness by a marked pain response causing the patient to withdraw from palpation because of pain. Mild lower facial asymmetry was apparent, with a slight deviation of the mandibular contour. The asymmetry was considered potentially related to asymmetric mandibular growth associated with TMJ involvement and was also observed, likely secondary to asymmetric mandibular condylar development, with associated mild hypoplasia of the left masseter muscle. Occlusal examination demonstrated an anterior open bite, with occlusal contacts limited to the first molars bilaterally.

**Figure 3:**
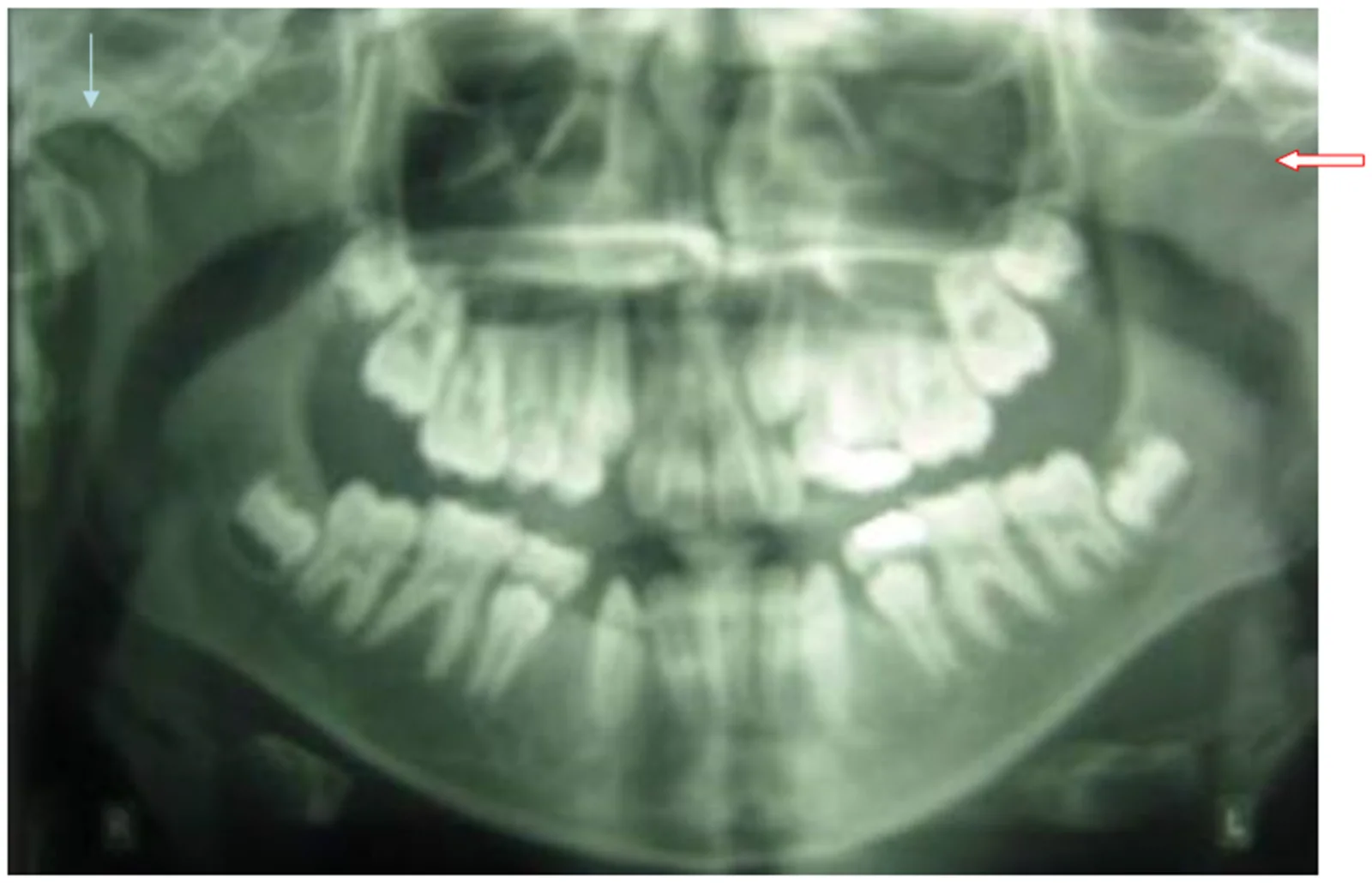
Panoramic radiograph of a female patient diagnosed with Juvenile Idiopathic Arthritis at the age of 12 years. In the right temporomandibular joint, the mandibular condyle (blue arrow) exhibits indistinct margins, irregular morphology, and a flattened, erosive articular surface, indicative of active bone degeneration. The left mandibular condyle (red arrow) is not adequately visualized, representing a limitation of the panoramic radiography that restricts detailed assessment of TMJ structural changes and may be attributable to a technical positioning error during image acquisition, precluding reliable evaluation of the left joint.

Panoramic radiography demonstrated mixed dentition and congenital agenesis of the mandibular first premolars (teeth 34 and 44), with no history of previous extraction. In the right TMJ, the mandibular condylar head exhibited indistinct cortical margins, possible osseous defects of the articular surface, irregular morphology, and a flattened erosive appearance (Figure 3).

**Figure 4:**
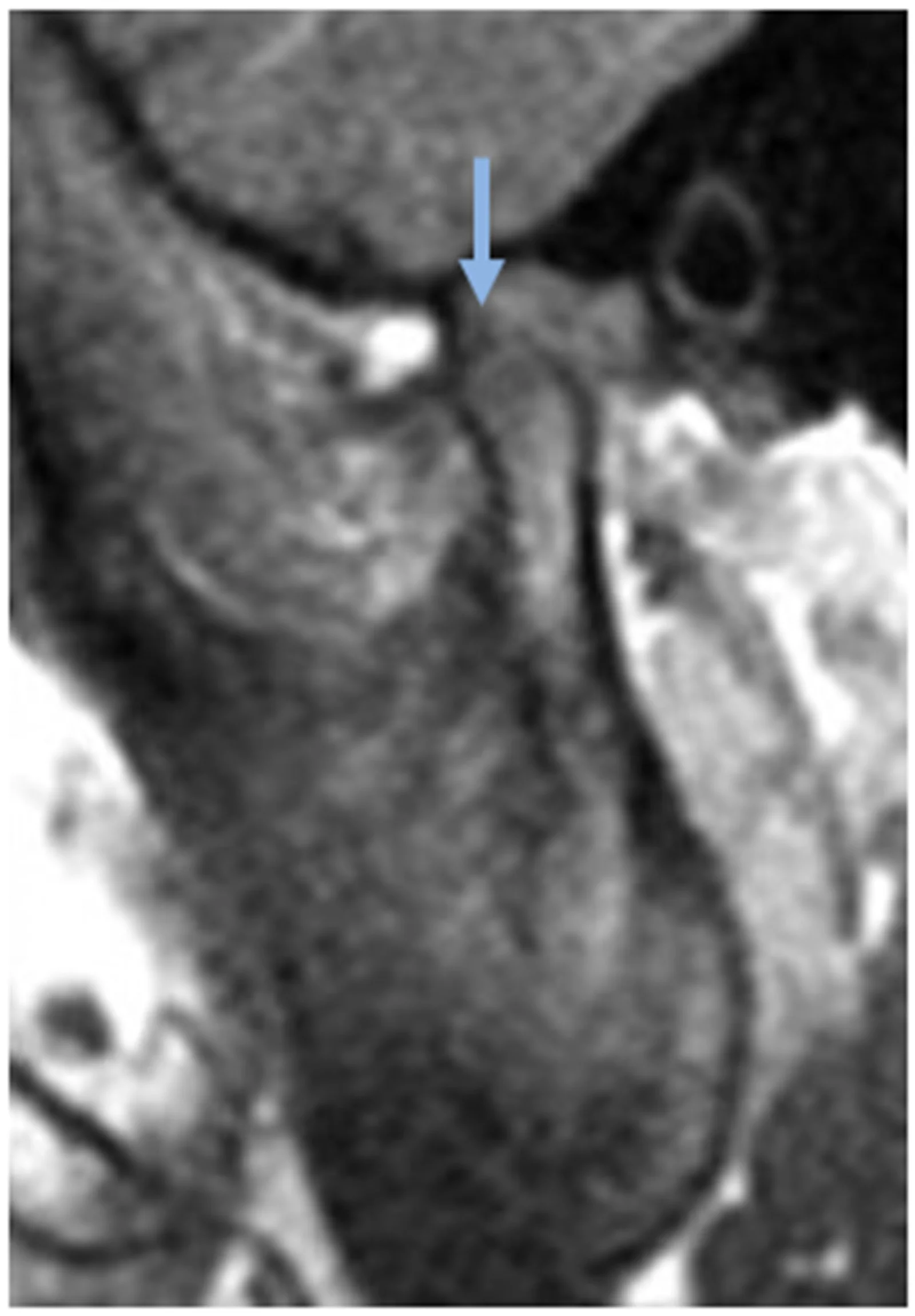
Magnetic resonance imaging of the viscerocranium in the sagittal plane, demonstrating the left temporomandibular joint of a female patient diagnosed with Juvenile Idiopathic Arthritis at the age of 12 years. The left temporomandibular joint (blue arrow) demonstrates mild condylar bone marrow edema, with imaging features suggestive of early erosive osseous changes. Anterior displacement of the articular disc without reduction during mouth opening is also observed, consistent with internal derangement.

**Figure 5:**
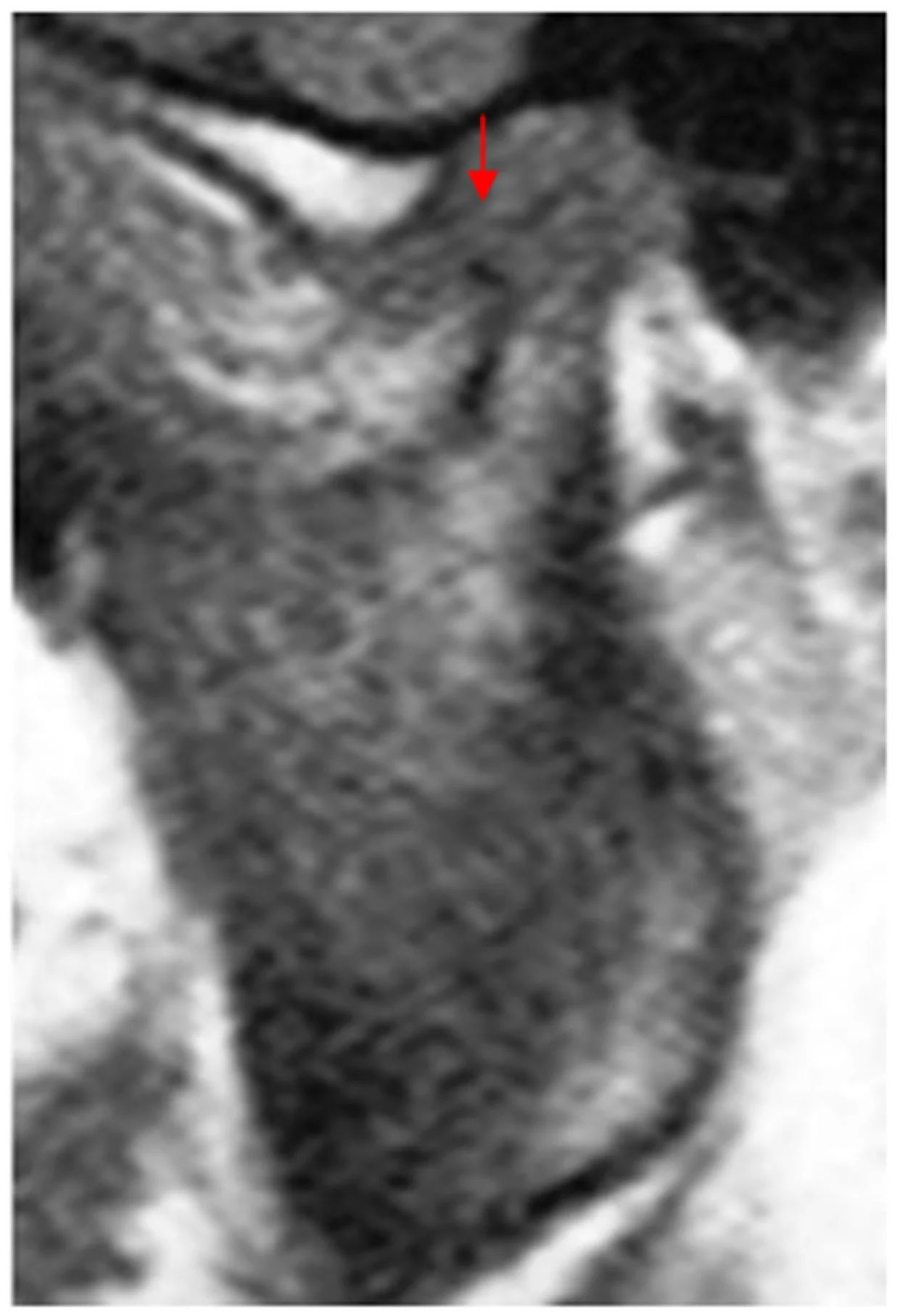
Magnetic resonance imaging of the viscerocranium in the sagittal plane, demonstrating the right temporomandibular joint of a female patient diagnosed with Juvenile Idiopathic Arthritis at the age of 12 years. The mandibular condyle exhibits a flattened articular surface with poorly defined cortical margins and features consistent with degenerative osseous changes. In the anterior aspect of the condyle (red arrow), a focal area of osseous apposition is observed, compatible with osteophyte formation.

MRI of both TMJs was performed in open- and closed- mouth positions, before and after intravenous contrast administration, in all three spatial planes. Bilateral synovial thickening with contrast enhancement of the TMJs was identified, consistent with active synovitis. The left mandibular condyle exhibited minimal bone marrow edema and early erosive alterations. The articular disc of the right TMJ was anteriorly displaced with reduction, whereas the disc of the left TMJ was diffusely thinned and anteriorly displaced without reduction (Figure 4 and Figure 5). These imaging findings were considered consistent with bilateral TMJ involvement secondary to JIA, as confirmed by the treating pediatric rheumatologist.

The Greek version of the KIDSCREEN-52 questionnaire was used to assess the patient’s health-related quality of life (HRQoL) at baseline, as well as at 1 and 6 months and 1 year after treatment initiation. The instrument assesses the frequency of feelings and behaviors and the intensity of attitudes using a five-point Likert scale (1 = never, 2 = seldom, 3 = sometimes, 4 = often, and 5 = always). KIDSCREEN-52 comprises 10 dimensions: physical well-being, psychological well-being, moods and emotions, self-perception, autonomy, parent relations and home life, peers and social support, school environment, social acceptance (bullying), and financial resources. Scores for each dimension are transformed to a 0–100 scale, with higher scores indicating better HRQoL. The patient’s KIDSCREEN-52 results are presented in Table 1 [23].

Based on clinical and imaging findings, a structured physiotherapy program was initiated, consisting of a systematic set of therapeutic exercises and controlled mandibular movements, aiming to increase mouth opening, reduce muscle tenderness, improve mandibular mobility, enhance muscle strength, and restore neuromuscular coordination. Physiotherapy, particularly when combined with occlusal splint therapy, may constitute a core component of conservative management of craniomandibular disorders in children with JIA. Mandibular stretching exercises were performed to the maximum pain-free range of opening and maintained for several seconds to progressively increase mandibular range of motion.

**Figure 6:**
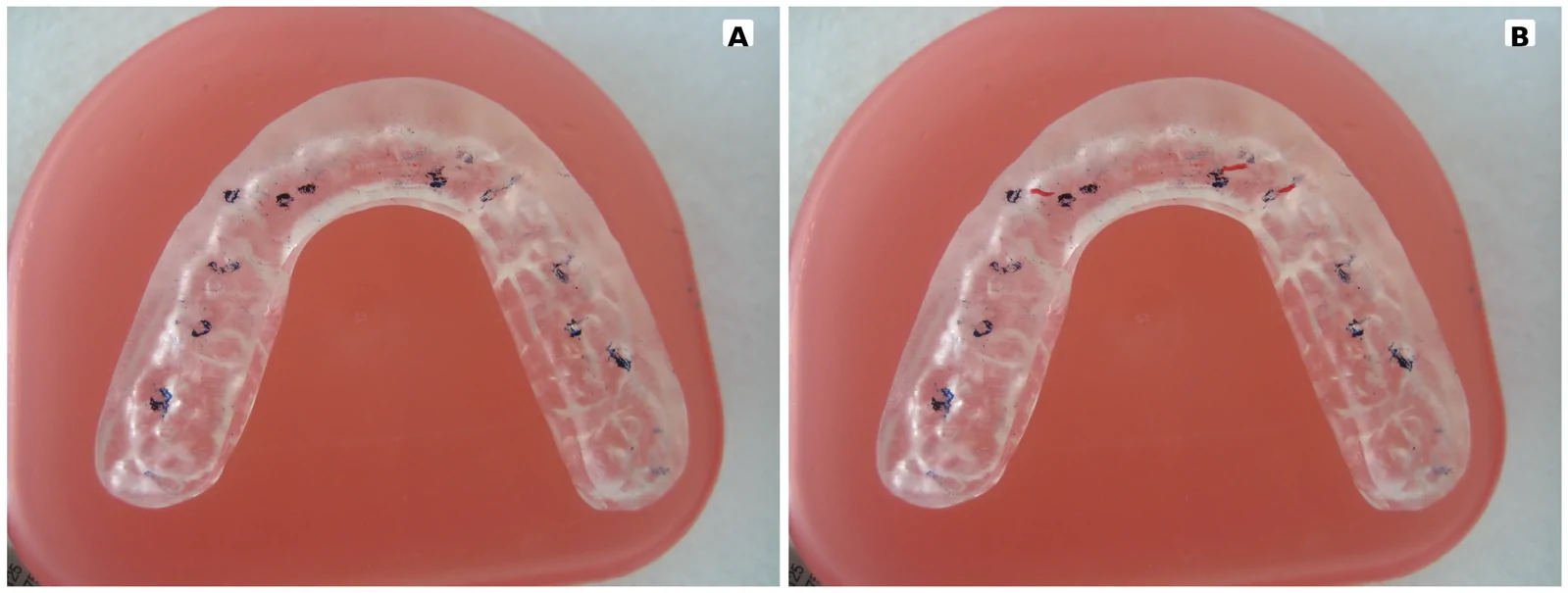
Patient-specific intraoral device (stabilization splint) used for the management of limited mandibular range of motion in a female patient diagnosed with Juvenile Idiopathic Arthritis at the age of 12 years. The stabilization splint is fabricated from heat-polymerized acrylic resin and features a flat occlusal surface with full dental coverage, without extension onto the gingival tissues or the palate. (**A**) In maximum intercuspation, uniform point contacts with the opposing dentition are established across the entire occlusal surface (blue dots). (**B**) During functional mandibular movements, including lateral excursions and protrusion, the splint is selectively adjusted according to the principles of mutually protected occlusion, ensuring appropriate disclusion and functional stability. The red articulating paper marks represent occlusal contacts recorded during excursive mandibular movements, including lateral and protrusive movements (red lines).

A maxillary stabilization splint was subsequently fabricated to maintain occlusal stability and reduce masticatory muscle hyperactivity (Figure 6). The splint was a removable, noninvasive orthopedic device, designed as a rigid flat stabilization splint, fabricated from heat-polymerized acrylic resin, with a soft internal lining to accommodate primary and erupting permanent teeth without exerting undesirable forces or interfering with tooth eruption. The splint covered all maxillary teeth to ensure optimal occlusal stability. Impressions of both dental arches and a maxillomandibular relationship record were obtained using reinforced bite-registration wax.

At delivery, the fit, retention, and occlusion of the splint were carefully evaluated and adjusted as necessary. The occlusal surface was maintained flat to permit smooth, unrestricted mandibular movements. Occlusal adjustment in maximum intercuspation was performed using articulating paper until uniform point contacts were achieved. Subsequent adjustments in eccentric positions were completed. During protrusive movements, anterior guidance resulted in posterior disclusion, in accordance with mutually protected occlusion principles. During lateral movements, canine guidance was established to disclude all other teeth. The patient was instructed to wear the splint exclusively during sleep. Compliance was excellent, with reported nightly use of approximately 8 h. One week after delivery, follow-up examination confirmed appropriate appliance use, improved mandibular mobility, and satisfactory splint adaptation.

At 3-month follow-up, the patient demonstrated substantial improvement in subjective symptoms, reporting a noticeable reduction in pain intensity and discomfort. Tenderness on palpation of the TMJs and masticatory muscles was significantly reduced, and maximum mouth opening increased from 26 mm at the initiation of the treatment to 41 mm. The intensity of the left TMJ sounds was also reduced. During this period, the patient continued disease-modifying antirheumatic drug therapy, including weekly injectable methotrexate and daily Filicin (vitamin B complex).

Regarding self-reported quality of life, the patient continued to experience pain in the lower extremities, particularly during running, resulting in reduced participation in physical activities and increased fatigue. Persistent pain in both radiocarpal joints, along with limited palmar flexion and occasional restriction of full elbow extension, also led to difficulties in activities of daily living and social activities (e.g., going out with friends). Headaches remained frequent, and psychological well-being continued to be impaired.

Six months after the initiation of the treatment, clinical improvement became evident, including reduced limitation of palmar flexion, significant improvement in mandibular range of motion and function, and a decrease in headache frequency from daily episodes to once per week. During this period, the patient reported complete resolution of orofacial pain symptoms, with mouth opening measured at 44 mm. Pediatric rheumatologic evaluation documented reduced limitation in palmar flexion and substantial improvement in TMJs, with minimal residual restriction.

The clinical improvement was further sustained at the 1-year follow-up. Maximum interincisal opening reached 50 mm, maximum mandibular protrusion was 7 mm, and lateral excursions measured 8 mm to the right and 6 mm to the left. No subjective stomatognathic symptoms were reported, and headache frequency decreased to 1–2 episodes per month. Throughout this period, rheumatologic examination revealed improved radiocarpal joint function, with only mild sensitivity on deep flexion. The patient also showed significant progress in self-reported quality of life, school performance, and physical activity.

A gradual splint-weaning protocol was subsequently implemented, leading to complete discontinuation of appliance use without recurrence of symptoms. After completing treatment at the COPM, the patient continued therapy with antirheumatic medications and remained under regular follow-up with a specialist rheumatologist. According to the patient’s report, orthodontic treatment was performed for approximately two years from the age of 14. Detailed orthodontic records, including the type of appliance, treatment mechanics, and post-treatment cephalometric or CBCT imaging, could not be obtained.

**Figure 7:**
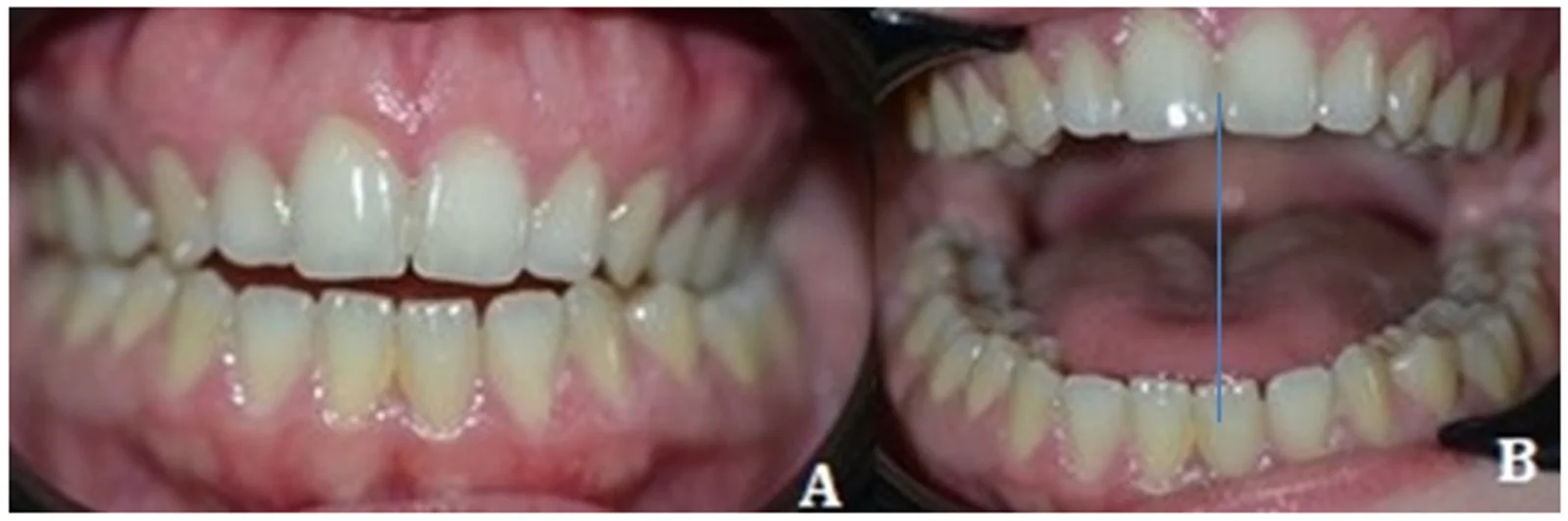
Clinical images of the mandible in both closed and open mouth positions in a female patient diagnosed with Juvenile Idiopathic Arthritis at the age of 27 years are presented. (**A**) In the maximum intercuspal position, there is a light anterior open bite, although the patient received orthodontic treatment for two years. (**B**) During maximal mandibular opening, a slight deviation of the mandible toward the right side is noted, measuring less than 2 mm (blue line). The maximum mouth opening was recorded at 46 mm, which falls within normal limits, indicating preserved mandibular mobility without clinically significant restriction. A change in mandibular deviation compared with the initial clinical presentation was observed; however, in the absence of detailed orthodontic records, it cannot be determined whether this change represented an intentional orthodontic correction.

During the COVID-19 pandemic, the patient discontinued the antirheumatic treatment. This led to a flare-up of symptoms, such as pain and difficulty moving the right elbow joint, as well as inability to achieve full extension. To manage the elbow stiffness, oral cortisone was administered, and after symptom remission, the patient continued pharmacological treatment with a csDMARD—methotrexate—for the management of symptoms of idiopathic arthritis. The patient also underwent orthodontic treatment for two years at the age of 14 (Figure 7).

After approximately 15 years of continuous antirheumatic treatment, including biologic agents, the 15-year follow-up was calculated from the initial diagnosis and assessment of TMJ involvement at 12 years of age; the patient had achieved a relatively stable clinical condition. The treating rheumatologist proposed a gradual reduction in the administered dosage, with the aim of minimizing long-term medication exposure while maintaining disease control. Following this treatment modification, the patient experienced a relapse of the disease-related symptoms, including pain and stiffness of the right upper-limb joints, accompanied by generalized musculoskeletal fatigue. The symptoms were reported to worsen with exposure to cold temperatures and during periods of increased psychological stress.

Fifteen years after the initial diagnosis of JIA, the patient remains under regular rheumatologic supervision and continuous long-term pharmacological treatment with biologic agents, specifically etanercept (Benepali^®^, Samsung Bioepis NL B.V., Delft, The Netherlands), a tumor necrosis factor-alpha (TNF-α) inhibitor classified as a biologic DMARD. Periodic physiotherapy sessions aimed at alleviating generalized musculoskeletal symptoms yielded limited improvement.

At the 15-year follow-up examination, clinical assessment of the stomatognathic system revealed tenderness on palpation of the left TMJ, as well as the masseter, temporalis, medial pterygoid, and floor-of-mouth muscles. Mandibular mobility remained functionally adequate, with a maximum mouth opening of 46 mm and mild deviation toward the right during opening. Lateral excursions were 7 mm to the left and 9 mm to the right. An anterior open bite persisted, accompanied by bilateral TMJ crepitus and mild headaches occurring approximately once per week.

**Figure 8:**
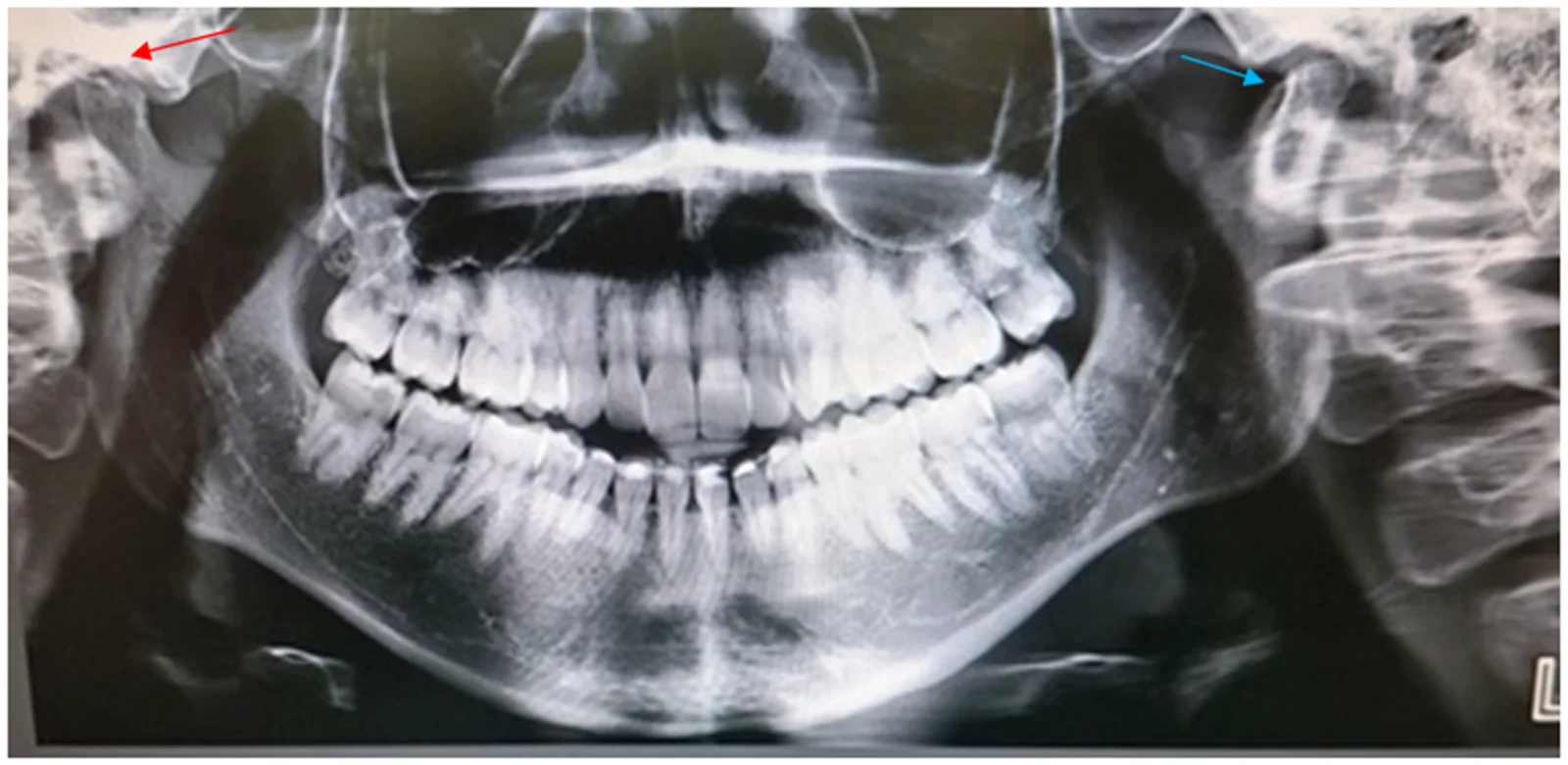
Orthopantomographic imaging of the patient at 27 years of age (a 15-year follow-up) reveals bilateral degenerative alterations of the temporomandibular joints, consistent with the chronic changes in the underlying condition and in agreement with the patient’s clinical findings. The left temporomandibular joint (blue arrow) demonstrates the presence of an intraosseous cyst along with osseous apposition on the condylar head, indicative of osteophyte formation and advanced degenerative remodeling. In contrast, the right temporomandibular joint (red arrow) exhibits flattening of the condylar head accompanied by a reduction in the interarticular space, as a result of the long-standing articular degeneration.

A follow-up panoramic radiograph demonstrated bilateral TMJ pathology suggestive of chronic structural changes. The left mandibular condyle exhibited an intraosseous cyst and osteophyte formation, whereas the right condyle demonstrated distinct flattening and altered morphology of the condyle head (Figure 8). Additionally, elongation of the lower facial third was observed, accompanied by persistent mild hypoplasia of the left masseter muscle (Table 2). These radiographic findings are compatible with chronic TMJ structural changes in the context of the patient’s long-term JIA and are consistent with the clinical findings observed at the 15-year follow-up.

## 4. Discussion

The primary therapeutic goal in JIA is to achieve inactive or minimally active disease in order to prevent joint damage, preserve function, and support normal growth and development. Current management relies on individualized therapeutic strategies combining DMARDs, biologic agents when indicated, and supportive non-pharmacological interventions, under the supervision of a pediatric rheumatologist [24].

TMJ involvement represents a particularly challenging manifestation of JIA because it may remain clinically inconspicuous despite underlying joint inflammation and structural abnormalities. TMJ arthritis may occasionally be the initial or sole manifestation of JIA and has been reported to be asymptomatic in approximately 65–85% of affected patients, potentially delaying recognition of joint involvement. When TMJ disease occurs during craniofacial growth, it may be associated with mandibular growth disturbances, facial asymmetry, malocclusion, pain, and functional impairment. Contrast-enhanced MRI is considered the reference imaging modality for detecting active TMJ inflammation and assessing early structural changes, although imaging findings should be interpreted in conjunction with clinical findings and longitudinal assessment [25].

The present case illustrates the complex relationship between clinical status and structural TMJ findings in a patient with JIA. During the initial treatment period, substantial improvement in pain and mandibular function was observed following optimization of systemic antirheumatic therapy together with adjunctive treatment consisting of an upper stabilization splint and a structured physiotherapy program. Maximum mouth opening increased from 26 to 46 mm, mandibular excursions improved to age-appropriate values, and masticatory function was restored. These findings are consistent with previous reports suggesting that occlusal splints and physiotherapy may provide symptomatic and functional benefits when incorporated into a multidisciplinary treatment strategy [26,27]. However, because systemic antirheumatic treatment was administered and modified during the same period, the individual contribution of splint therapy or physiotherapy to the observed clinical improvement cannot be determined from this single case. The clinical response should therefore be interpreted as the outcome of the overall multidisciplinary and concomitant therapeutic approach rather than attributed to any individual intervention.

Additionally, the congenital agenesis of teeth 34 and 44 found on panoramic radiography may have contributed to altered occlusal stability and dentoalveolar development; however, it was considered a potential contributing factor rather than a primary cause of the observed facial asymmetry, which was more likely related to TMJ involvement and altered mandibular growth. Against this background, the 15-year longitudinal follow-up demonstrated that substantial and sustained improvement in TMJ function may coexist with persistent or progressive structural changes, underscoring the limitations of clinical assessment alone and highlighting the need for long-term imaging surveillance in patients with JIA-related TMJ involvement.

An important observation emerged from the long-term follow-up. Fifteen years after the initial presentation, the patient remained free of pain and demonstrated excellent mandibular function; however, panoramic radiography revealed bilateral condylar morphological abnormalities, including flattening, osteophyte formation, and intraosseous cystic changes. These findings demonstrate that substantial structural abnormalities may be present in a patient with JIA-associated TMJ involvement despite a favorable long-term clinical status. Nevertheless, an important methodological limitation must be acknowledged. The initial assessment was performed using contrast-enhanced MRI, whereas the long-term assessment was based on panoramic radiography. Because these imaging modalities differ substantially in their sensitivity, anatomical representation, and ability to characterize inflammatory and structural joint changes, the findings cannot be directly compared to establish longitudinal structural progression. In particular, the available data do not permit determination of when the observed radiographic abnormalities developed or whether they represent continuous progression from the initial TMJ findings. The long-term findings should therefore be interpreted as evidence of persistent or later-detected structural abnormalities rather than definitive evidence of progressive structural deterioration.

This distinction is clinically important because the absence of pain and restoration of mandibular function do not necessarily provide sufficient information about the structural status of the TMJ. Conversely, structural abnormalities identified on imaging do not necessarily imply current clinical dysfunction. The present case therefore illustrates a potential dissociation between clinical adaptation and structural joint status, while also highlighting the limitations of drawing conclusions about disease trajectory from non-comparable imaging examinations. Our findings are consistent with those of Twilt et al., who emphasized that the temporomandibular joint is difficult to assess clinically and that appropriate radiological examination is essential for accurate diagnosis and treatment assessment in patients with JIA [28]. Appropriate clinical surveillance, with imaging guided by the clinical context and the suspected disease activity or structural consequences, may be warranted in patients with a history of TMJ involvement, particularly during periods of craniofacial growth.

Current evidence supports conservative dental interventions as adjunctive rather than primary treatments for TMJ involvement in JIA. Occlusal splints, physiotherapy, and therapeutic jaw exercises may contribute to pain reduction and improvement of mandibular function and quality of life; however, high-quality pediatric evidence remains limited, and their capacity to modify the underlying inflammatory process or prevent craniofacial growth disturbances has not been established [29,30]. Similarly, local interventions, including arthrocentesis and intra-articular corticosteroid injections, should be considered selectively and following multidisciplinary evaluation, given the limited evidence regarding their long-term effects on the developing TMJ [31].

The management of JIA-associated TMJ involvement therefore requires close collaboration among pediatric rheumatologists, pediatric dentists, orthodontists, oral and maxillofacial radiologists, oral and maxillofacial surgeons, physiotherapists, and orofacial pain specialists. Integration of systemic disease control with appropriate TMJ assessment, functional rehabilitation, individualized dental management, and longitudinal clinical surveillance is particularly important in growing patients.

Several limitations of the present case should be explicitly acknowledged. First, this is a single-case report, and the observations cannot be generalized to the broader JIA population. Second, the long-term follow-up included gaps in clinical and imaging documentation, limiting the ability to reconstruct the course of TMJ disease continuously over the intervening years. Third, standardized serial TMJ imaging was not available throughout follow-up. Most importantly, the baseline and long-term assessments used different imaging modalities, namely contrast-enhanced MRI and panoramic radiography, respectively, precluding direct quantitative or qualitative assessment of structural change over time. Finally, systemic antirheumatic treatment and supportive management changed over the course of follow-up, making it impossible to determine the independent contribution of any particular therapeutic intervention to the observed long-term outcome. These limitations should be considered when interpreting the apparent discrepancy between the patient’s excellent clinical status and the structural abnormalities identified at long-term follow-up.

## 5. Conclusions

TMJ involvement in JIA may be clinically inconspicuous despite the presence of structural abnormalities, particularly during periods of craniofacial growth. This long-term follow-up case demonstrates that substantial condylar abnormalities may be identified in adulthood despite sustained absence of pain and excellent mandibular function. However, because the initial and long-term assessments were performed using different imaging modalities and standardized serial imaging was unavailable, the present case does not establish continuous or progressive structural deterioration over the 15-year period.

Conservative interventions, including occlusal splint therapy and physiotherapy, may provide meaningful improvements in pain and mandibular function as adjuncts to systemic treatment, but their independent therapeutic contribution cannot be established from this case, and they should not be considered substitutes for adequate control of the underlying disease. The findings support the importance of individualized multidisciplinary management and appropriate longitudinal clinical assessment of patients with JIA-associated TMJ involvement. Future prospective multicenter studies incorporating standardized clinical assessments and comparable serial TMJ imaging are needed to better characterize structural and functional trajectories, identify patients at increased risk of clinically important TMJ involvement, and establish evidence-based monitoring and management strategies.

## Figures and Tables

**Table 1:** Treatment History and Clinical Course of the Patient with Juvenile Idiopathic Arthritis over a 15-Year Period.

Age-Date Follow-Up (FU)	Clinical Findings	Stomatognathic System Findings	Pain NRS	KIDSCREEN-52	Treatment
Age 12, (Baseline) October 2011	Left knee: swelling with mild effusion and pain during running. Left hand: tendon thickening and wrist effusion. Right hand: minimal peritendinous fluid.	Orofacial pain, restricted mouth opening, mandibular deviation. Left TMJ sounds.	8	PWB: 56; PSC:58; MOOD:63; SP:65; AUT:59; PRHL:79; PSS:71; SCH:65; SA:90; FR:73	Intra-articular corticosteroid injections to the left knee and wrist; prednisolone 20 mg/day; oral MTX 20 mg/week (8 tablets/week, assuming 2.5-mg tablets); adalimumab (Humira^®^), dose not documented
November 2011	Hyperplasia and mild tenderness of left carpal joint capsule.	Bilateral TMJ pain. Muscle tenderness. MMO: 26 mm.	8		MTX 12.5 mg/week; prednisolone 5 mg/day; folinic acid; alfacalcidol; adalimumab (Humira^®^), dose not documented
December 2011	Mild lower extremity and carpal joint pain.	Left TMJ clicking; muscle tenderness. MMO: 40 mm.	8		Splint; MTX; Prednisolone; folinic acid; alfacalcidol
February 2012	Mild knee pain; persistent carpal discomfort.	Reduced muscle tenderness; MMO: 41 mm.	6		Splint; MTX; folinic acid; alfacalcidol
March 2012 (3-month FU)	Synovial hyperplasia and effusion; frequent headaches.	Masseter and temporalis tenderness; MMO: 41 mm.	5	PWB: 62; PSC:60; MOOD:66; SP:67; AUT:60; PRHL:80; PSS:71; SCH:66; SA:91; FR:73	Splint; Metoject (methotrexate) 15 mg/week SC
June 2012	Bilateral carpal capsule hyperplasia; mild pain; reduced flexion.	No tenderness; TMJ clicking; MMO: 44 mm.	2	PWB: 71; PSC:70; MOOD:74; SP:68; AUT:63; PRHL:81; PSS:72; SCH: 70; SA:91; FR:75	Splint; Metoject 15 mg/week SC; folinic acid; alfacalcidol
(6-month FU)					
October 2012	Improvement; no capsule hyperplasia; mild pain.	No tenderness; TMJ clicking; MMO: 50 mm.	1	PWB: 73; PSC:74; MOOD:77; SP:70; AUT:72; PRHL:82; PSS:74; SCH: 75; SA:92; FR:75	Splint; Metoject 15 mg/week SC; folinic acid; iron; alfacalcidol
(1-year FU)					
March 2013	Asymptomatic joints.	No tenderness; TMJ clicking; MMO: 50 mm.	0		Splint discontinued; Metoject 15 mg/week SC; folinic acid
August 2013	Clinical remission.	Asymptomatic.	0		MTX/Metoject discontinued; biologic DMARD therapy—exact agent and dose should be specified only if documented in the medical record
2013–2015	-	-	-		Orthodontic treatment
2020 (COVID-19)	Right elbow pain; reduced ROM.	-	-		Biologic DMARD discontinued; hydrocortisone
Age 27	Right elbow pain; fatigue.	TMJ and muscle tenderness; MMO: 46 mm.	3		Etanercept 50 mg/week SC; folinic acid; vitamin D
January 2026					

Abbreviations: MMO = Maximum mouth opening; MTX = Methotrexate; SC = Subcutaneous; TMJ = Temporomandibular joint; NRS = Numeric Rating Scale; Splint = patient-specific intraoral device; PWB = Physical well-being; PSC = Psychological well-being; MOOD = Moods and emotions; SP = Self-perception; AUT = Autonomy; PRHL = Parent relations and home life; PSS = Peers and social support; SA = Social acceptance (bullying); SCH = School environment; FR = Financial resources; FU = follow-up.

**Table 2:** Longitudinal Patient Findings Mapped to the Six Items of the Short Clinical Orofacial Examination Protocol for Juvenile Idiopathic Arthritis.

Examination (6-Items)	Baseline	6-Month FU	1-Year FU	15-Years FU
Clinician-assessed pain location	Pain localized to left TMJ, deep pressure-like pain, daily frontal headaches	Complete resolution of orofacial pain	No stomatognathic pain; headaches reduced to 1–2/month	Mild weekly headaches; persistent TMJ and masticatory muscle tenderness (left predominance)
TMJ pain on palpation (closed mouth)	Bilateral TMJ tenderness, left > right	Minimal residual TMJ sensitivity; substantial improvement	No pain reported	Tenderness on left TMJ and masticatory muscles
TMJ pain on palpation (open mouth)	Pain during mandibular function, left > right	Pain fully resolved during function	No pain reported	Functional movement with residual discomfort/tenderness; bilateral TMJ crepitus present
Mandibular deviation (≥3 mm)	Left-sided deviation during opening	Significant improvement in mandibular control	No clinically relevant deviation reported	Mild right-sided deviation during opening
Maximal mouth opening	26 mm (severely restricted)	44 mm	50 mm	46 mm (normal functional range preserved)
Frontal facial asymmetry	Left-sided mild lower facial asymmetry. The asymmetry was considered potentially related to asymmetric mandibular growth associated with TMJ involvement due to asymmetric condylar development; mild left masseter hypoplasia	No change reported	No change reported	Persistent asymmetry: elongation of lower facial third + mild left masseter hypoplasia
Facial profile classification	Not reported	Not reported	Not reported	Not reported

TMJ: temporomandibular joint; FU = follow-up.

## Data Availability

The original contributions presented in this study are included in the article. Further inquiries can be directed to the corresponding authors.
